# Neural Correlate of Filtering of Irrelevant Information from Visual Working Memory

**DOI:** 10.1371/journal.pone.0003282

**Published:** 2008-09-26

**Authors:** Shahin Nasr, Ali Moeeny, Hossein Esteky

**Affiliations:** 1 School of Cognitive Sciences, IPM, Tehran, Iran; 2 Research Center for Brain and Cognitive Sciences, School of Medicine, Shaheed Beheshti University, Tehran, Iran; 3 Neuroscience Research Center, Shaheed Beheshti University of Medical Sciences, Tehran, Iran; Victoria University of Wellington, New Zealand

## Abstract

In a dynamic environment stimulus task relevancy could be altered through time and it is not always possible to dissociate relevant and irrelevant objects from the very first moment they come to our sight. In such conditions, subjects need to retain maximum possible information in their WM until it is clear which items should be eliminated from WM to free attention and memory resources. Here, we examined the neural basis of irrelevant information filtering from WM by recording human ERP during a visual change detection task in which the stimulus irrelevancy was revealed in a later stage of the task forcing the subjects to keep all of the information in WM until test object set was presented. Assessing subjects' behaviour we found that subjects' RT was highly correlated with the number of irrelevant objects and not the relevant one, pointing to the notion that filtering, and not selection, process was used to handle the distracting effect of irrelevant objects. In addition we found that frontal N150 and parietal N200 peak latencies increased systematically as the amount of irrelevancy load increased. Interestingly, the peak latency of parietal N200, and not frontal N150, better correlated with subjects' RT. The difference between frontal N150 and parietal N200 peak latencies varied with the amount of irrelevancy load suggesting that functional connectivity between modules underlying fronto-parietal potentials vary concomitant with the irrelevancy load. These findings suggest the existence of two neural modules, responsible for irrelevant objects elimination, whose activity latency and functional connectivity depend on the number of irrelevant object.

## Introduction

We live in a dynamic environment where object relevancy to the task in hand could be altered through the time making some potentially useful stimuli irrelevant to the task in a later stage. Frontal cortex has been shown to be critical in blocking irrelevant information from entering working memory (WM) when their irrelevancy is clear from the beginning of the task [1]–[3]. However, it is not always possible to dissociate relevant and irrelevant objects from the very first moment they come to our sight. In this condition, any imprudent filtering could impair subjects' performance by eliminating relevant items from further processing. Here, subjects need to retain maximum possible information in their WM until it becomes clear which items should be eliminated from WM to free attentional and memory resources. The mechanism underlying this type of irrelevancy elimination from WM is not known yet.

The role of frontal and parietal cortices respectively in information retention and distracter filtering is well established. Recent studies have shown that parietal activity in memory maintenance period is correlated to the amount of WM load [4]–[6] while frontal activity during is shown to be necessary to prevent distracters from entering WM when human subjects are involved in two interleaving, main and distracting, spatial memory tasks [2]. In these experiments and other similar studies [1], [3], relevant and irrelevant items have been clearly dissociated from each other from the very beginning of the task. Thus, activities in frontal and other brain areas reported in these studies are an indication of their involvement in preventing irrelevant items from entering WM. In such studies parietal involvement in distracter filtering seems to be unnecessary since irrelevant items had been eliminated before reaching parietal cortex which is responsible for retention of items in WM [4]–[5]. In contrary, when the irrelevant items in addition to the relevant ones are already stored in WM, parietal involvement in irrelevancy elimination might be crucial. In this condition, in addition to the frontal cortex, parietal cortex could be a part of irrelevancy filtering network.

An alternative view to irrelevancy filtering has been put forward by some authors [7]–[9] suggesting that biasing attentional resources toward relevant objects, called selection process, is responsible for irrelevancy suppression. According to this hypothesis feedback signal, mainly from frontal cortex, biases the competition between relevant and irrelevant items in favour of the task relevant items [7]–[9]. Thus, in a selection process the number of relevant items and the time needed for their detection and biasing attention towards them mainly influence the processing duration. In contrast to this selection procedure, in a filtering mechanism in which irrelevant items are directly detected and inhibited, processing duration is proportional to the number of irrelevant items.

To explore the brain mechanism for elimination of irrelevant items from WM (filtering vs. selection) and its neural correlates, we examined brain ERP activities of normal human subjects, during a modified version of change detection task. In this task the relevancy or irrelevancy of sample items were revealed to the subjects after a delay, when test stimuli were presented, forcing the subjects to store all of the sample items in WM. In order to understand the mechanism underling irrelevancy elimination we controlled the amount of relevancy and irrelevancy loads independent from each other as they are two indexes representing selection and filtering processes, respectively. We found that subjects' RT and ERP latency depended on the amount of irrelevant objects number and therefore, filtering and not selection mechanism is responsible for eliminating irrelevant information from visual WM.

## Methods

### Participants

Fourteen healthy male undergraduate students (age range: 20–28 years) were paid to participate in this experiment. None of the participants had any history of neurological or psychiatric disorders. Participants had normal or corrected to normal visual acuity. The experiment was approved by the Shahid Beheshti Medical University ethics committee and Iranian Society for Physiology and Pharmacology and subjects gave written informed contest before the experiments when all procedures were explained to them.

### Stimuli and Procedure

**Figure 1:**
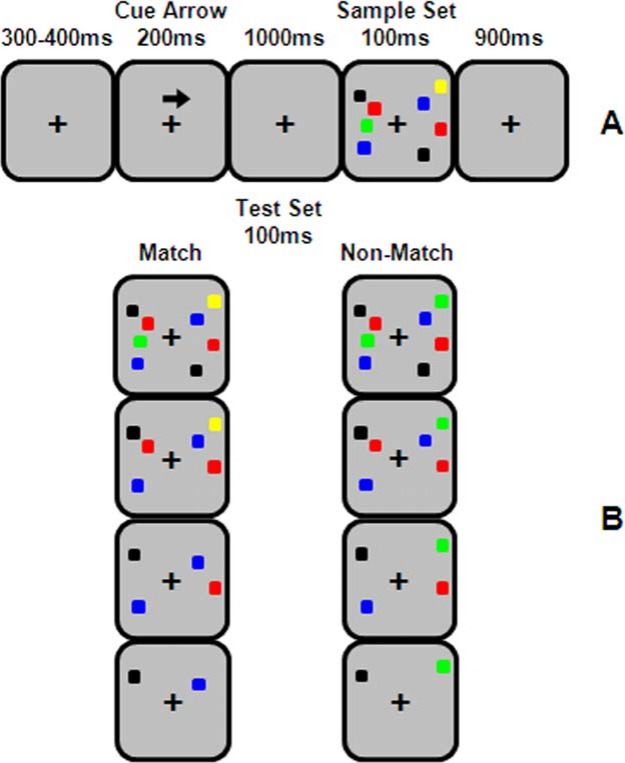
Example of stimuli and experiment procedure. (a) *The sequence of cue and sample presentati*on. Participants had to retain the sample *objects in th*e cued hemifield for 900ms during blank interval and then compare it with the test set to find the potential colour discrepancy. (b) An example of a test set corresponding to the presented sample in part a. Participants had to report any colour discrepancy between sample and test sets while they had to ignore any change due to object elimination. The first row corresponds to whole report trials while other rows demonstrate partial report trials with different number of objects within the test set. Left and right columns represent examples of match and non-match conditions, respectively.

In this study we used a procedure partially similar to the one used previously by Vogels & Machizawa (2004). In this procedure each trial started by presenting a fixation cross in the center of the screen. After 300–400ms a cue arrow was presented above the fixation cross. Direction of the arrow indicated that objects presented in the pointed visual hemifield should be memorized and that the objects in the other hemifield should be ignored. The fixation cross remained on screen during the rest of trial but the arrow was removed after 200ms. After a 1000ms interval, two arrays of sample stimuli were presented. Each array was presented within a 4°×7.3° rectangular regions in each visual hemifield. These regions were centred 3° from the fixation cross and consisted of 2–7 coloured squares. Each coloured square subtended 0.65°×0.65° of visual field and its colour was selected randomly from a set of seven highly discriminable colours (black, white, red, green, blue, yellow & pink). A particular colour was not selected more than two times in each hemifield. All stimuli were presented on a light gray background (30cd/mm^2^). Positions of the coloured squares within the rectangular regions were randomized in each trial with the constraint that the distance between each two squares be at least 2° (centre to centre). In each trial the number of items in the left and right hemifields was identical but location and colour of the presented items could vary between the hemifields. We presented sample stimuli for 100ms and participants had to memorize presented objects in the cued hemifield to compare them later with test stimuli. The test stimuli were presented 900ms from the sample offset (Figure 1a).

The most prominent difference between our procedure and the one used by Vogel & Machizawa (2004) was in the test set. In their procedure, subjects participated in a whole report (WR) paradigm. In their paradigm, the number of objects within sample and test sets was always the same and all the presented items within sample set were required and relevant to the task. In our experiment, in addition to WR paradigm, in some trials we randomly used partial report (PR) paradigm. In this condition, the number of objects in the test set was smaller than those in the sample set. In both trial types, participants had to report whether there was a colour discrepancy (50% of trials) between the test set objects and their corresponding objects within the sample. In PR trials participants should ignore any change regarding a potential difference between the number of objects in the sample and test sets and they had to confine their colour comparison to those objects presented in both sets. In this condition the eliminated sample objects became irrelevant to the task (Figure 1b).

In this experiment all possible combinations of relevancy and irrelevancy loads were presented with equal frequency. Thus in contrast to previous studies that confined PR to cueing or presenting just one object in the test set [10]–[11], in our paradigm, number of objects within the test set varied across different trials. Altogether 77.8% (21/27) of trials were PR and 22.2% (6/27) were WR. The sequence of trials was randomized so participants did not know whether a trial was WR or PR until the test image was presented. It is noteworthy that in this condition relevancy (number test objects number) and irrelevancy loads (the number of eliminated) varied independently. For example, when relevancy load was equal to one, the irrelevancy load still could varied between one to six corresponding to the trials with two to seven objects within the sample sets.

During the experiment, accuracy and speed were both stressed. We accepted those responses within 1500ms after test onset and few trials with longer response time were eliminated from the results. Subsequent trials started 1800ms after the test offset. Before the experiment, subjects participated in a few training trials (<50 trials) to become familiar with the task. Participants' responses during these trials were not included in results. Each subject participated in 1260 trials within 15 blocks with 2 minutes break between them. Two participants with more than 20% eliminated trials (due to late response) were excluded from the experiment.

### ERP Recording

EEG recording were made by using a Neuroscan system with 32 Ag/AgCl sintered electrodes mounted on an elastic cap. Data were acquired continuously in AC mode (0.05–30Hz) with 1 kHz sampling rate. Reference electrodes were linked mastoids, grounded to AFz. Four electrodes monitored horizontal and vertical eye movements for off-line artifact rejection. Electrodes impedance were kept <5kΩ. Data was resampled off-line by 250Hz sampling rate. Baseline was corrected on the basis of activities, recorded 100ms before the sample stimulus onset. A separate analysis was applied in order to eliminate those trials with eye movement and eye blinks, during100ms before the sample onset up to 800ms after the test onset, by detecting those trials on which the peak-to-peak voltage in the horizontal and vertical eye movement channels exceeded 30 µV (<10% of trials).

Here we divided cortical activities into four groups according to their spatial distribution and participants brain activity mapping; 1) frontal leads (recorded by FP1, F3, F7, FC3, Fz, FCz, FP2, F4, F8 and FC4 sensors), 2) parietal leads (recorded by P3, P7, CP3, P4, P8, CP4, CPz and Pz sensors), 3) occipital lead (recorded O1, O2 and Oz sensors) and, 4) temporal leads (recorded by T7, T8, TP7, TP8, FT7 and FT8 sensors). We only analyzed the ERP activities after test onset because earlier activities are shown to be correlated with memory retention [5]–[6], [12] rather than those processes related to irrelevancy filtering or change detection.

### Data Analysis

For each experimental condition averaged ERP signal was calculated for each participant separately. Grand averaged ERPs were calculated by averaging individual participants' signal. In order to examine the effects of experimental parameters on frontal and parietal activities we measured ERP activity using a sliding window. In this method, we divided the first 800ms of ERP activities, which was recorded after onset of test object set into separate time windows with 48ms length and 36ms overlap between each two adjacent windows. In each time window, we applied a factorial ANOVA to ERP area under curve. Using this method enabled us to detect any modulation in ERP activities related to experimental conditions with very low onset time estimation error (<12ms).

## Results

### Participants Behavior

**Figure 2:**
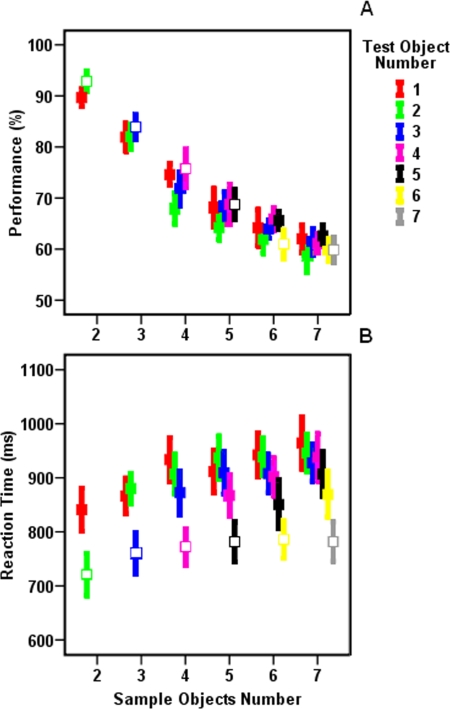
Participants' response accuracy (a) and RT (b) in different experimental conditions. Participants' performance declined as the number of sample objects increased (*p*<0.001) while participants' RT was mainly affected by the trial type (*p*<0.001). In both graphs open and closed squares correspond to whole report and partial report trials respectively. Error bars represent one standard error.

We checked the effect of number of sample objects and trial type on participants' response accuracy and RT by applying two separate two-factor ANOVA (figure 2a). Consistent with the previous studies of visual WM [10]–[11], [13] we found that increasing the number of objects in sample set significantly reduced participants' response accuracy (F(5,132) = 52.75, *p*<0.001). We did not find any significant difference between participants' response accuracy during partial report (PR) trials that needed irrelevancy elimination and whole report (WR) trials that did not need any elimination (F(1,132) = 0.66, *p*>0.05). The interaction between the two factors also remained non-significant (F(5,132) = 0.87, *p*>0.05). Lack of trial type effect indicates that participants' response accuracy depends on the amount of memory load (i.e. sample objects number) and reducing the number of objects within the test set does not improve their response accuracy.

Despite the fact that participants' response accuracy remained insensitive to trials type, it was still possible that participants used excessive processes during PR or WR trials, relative to the other one, which consequently affected participants' RT. In order to examine this possibility we applied a two-factor ANOVA (sample objects number and trials type) to participants' RT (figure 2b). In contrast to participants' response accuracy, we found that participants' RT was significantly (F(1, 132) = 17.06, *p*<0.001) different between trials that required irrelevancy elimination (i.e. PR trials) and those that did not require any irrelevancy elimination (i.e. WR trials). Here, participants showed significantly shorter RT during WR trials (767±123ms) compared to PR trials (905±132ms). The effect of sample objects number (F(5, 132) = 1.20, *p*>0.05) and its interaction with trial type (F(5, 132) = 0.06, *p*>0.05) remained non-significant.

**Figure 3:**
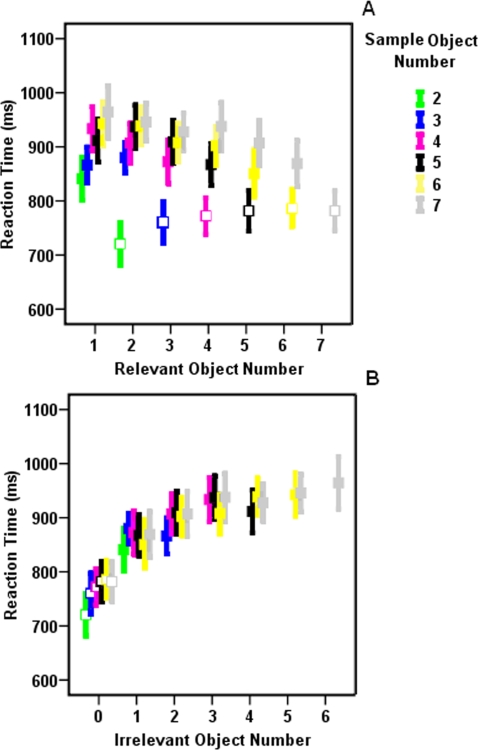
The relation between participants' RT and the number of (a) relevant and (b) irrelevant objects. Pearson test of correlation showed that there was a significant correlation between participants' RT and the number of relevant (r = −0.204, *p*<0.001) and irrelevant (r = 0.401, *p*<0.001) objects. Importantly, subsequent Pearson test for comparing two correlation coefficients yielded that participants RT is significantly better correlated to irrelevant objects number (*p*<0.01). Open and closed squares correspond to whole report and partial report trials, respectively. Error bars represent one standard error.

We further checked the effect of two experimental parameters on subjects' RT: (1) number of objects within the test set which represents the amount of relevancy load (figure 3a) and, (2) amount of difference between objects quantity in the sample and test sets which represents the amount of irrelevancy load (figure 3b). Since sum of relevant and irrelevant objects numbers should be equal to the sample objects number, we could not generate trials with all combinations of these two values (e. g. we could not generate a trial with 6 relevant and 4 irrelevant objects number since it needs 10 objects within the sample set and our sample set always contained less than 8 objects). Therefore we could not assess the effect of these two experimental parameters on subjects' RT by a single application of two-way ANOVA. Here, we used three different tests to assess the effect of experimental parameters on subjects' RT. First, we confined our analysis to a subset of trials in which all combinations of relevant and irrelevant objects number existed (i.e. trials in which relevant and irrelevant objects number was less than 4). In these trials, application of two-factor ANOVA (relevant and irrelevant objects number) yielded a significant effect of irrelevant objects number (F(2, 99) = 10.779, *p*<0.001) without any significant effect of relevant objects number (F(2, 99) = 0.336, *p*>0.05) or interaction between the two factors (F(4, 99) = 0.350, *p*>0.05).

Second, In order to use a larger subset of trials for assessing the effect of experimental parameters, we repeated the application of two-factor ANOVA but here we used trial types (WR trials vs. PR trials) and number of test objects as independent parameters. This test enabled us to assess whether number of test objects (i.e. selection load) was responsible for subjects' RT variation or objects elimination between sample and test sets. In this test, trials with either one or seven objects within test set were excluded since these trials were always PR and WR respectively and there was no alternative trial types for them. Application of two-way ANOVA (test objects number vs. trial type) yielded that subjects' RT was significantly longer during PR trials compared to WR trials (F(1,110) = 42.67, *p*<0.001). While the effect of test objects number (F(4,110) = 0.08, *p*>0.05) and the interaction between the two factors (F(4,110) = 1.07, *p*>0.05) remained non-significant. Thus, eliminating irrelevant objects between sample and test sets and not the exact number of test objects affected subjects' RT.

Third, we measured the predictability of subjects' RT on the basis of relevant (i.e. number of test objects), irrelevant objects (i.e. number of objects eliminated between sample and test) and also sample objects numbers by measuring the correlation between participants' RT and these three factors. Here we used all data trials rather than a subset of trials. Applying three separate Pearson tests of correlation indicated that participants' RT showed the highest correlation with the irrelevancy load (*p*<0.001, r = 0.401). We also found correlation between the number of sample objects (*p*<0.001, r = 0.215) and the number of test objects (*p*<0.001, r = −0.204) with participants' RT but their correlation was not comparable to the correlation between number of irrelevant objects and participants' RT. Applying subsequent Pearson test for comparing correlation coefficients, we found that the difference between correlation coefficients was significant: participants' RT was significantly more correlated to the amount of difference between sample and test sets (*p*<0.01).

Showing that participants' RT increase as we eliminate more objects between sample and test sets also rules out the possibility that shifting attention between test objects number, to find the possible change location, is responsible for delayed participants' RT. Remembering the eliminated items is also unlikely to be responsible for such delay since in trials with numerous eliminated objects this process could be highly erroneous whereas participants' response accuracy did not show any impairment in PR trials. The only possible reason for the increased participants' RT seems to be WM re-organization by filtering of task irrelevant objects. Here, rather than remembering the eliminated objects, irrelevant objects are actively suppressed from WM. Using this mechanism enables participants to free memory and attentional resources, already used by irrelevant objects, for further decision making processes.

### Participants' ERP

In previous section we showed that participants' RT increased as we increased the number of eliminated objects between sample and test sets (i.e. irrelevancy load) suggesting that participants used a filtering process to eliminate irrelevant objects from their WM. In order to understand neural correlates underlying this filtering process we assessed ERP brain activities of 12 human participants when they were performing the change detection task. As mentioned in the method section we only analyzed the ERP activities after test onset because filtering process started only when the irrelevant objects were revealed. Much of the results presented here concerns two ERP potentials observed in frontal (N150) and parietal (N200) cortices since they were found to be tightly correlated with different aspects of the filtering mechanism used to eliminate irrelevant objects.

**Figure 4:**
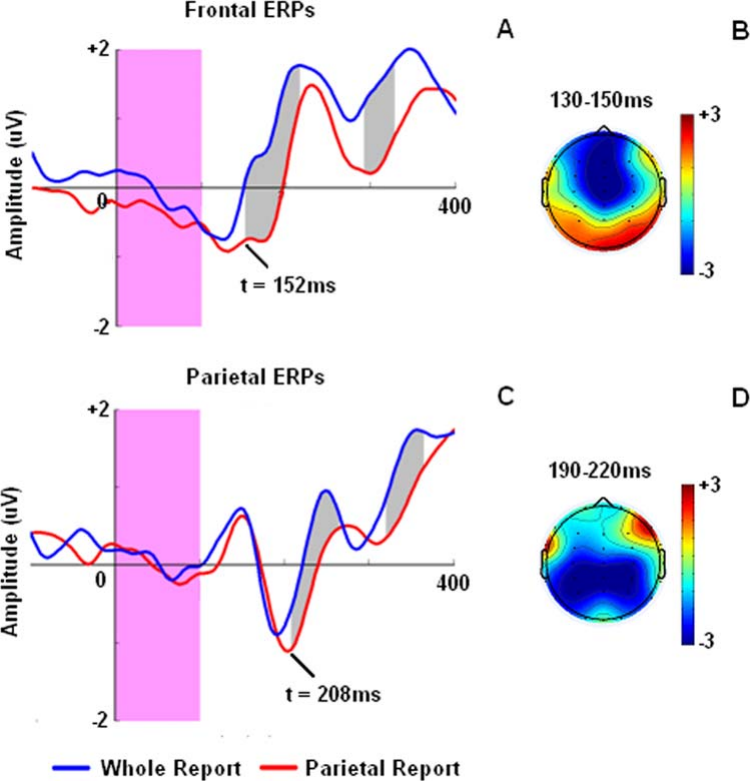
Early (first 400ms) ERP activities recorded in frontal (a) and parietal (c) leads after test onset. Activity mappings also demonstrate distribution of frontal (b) and parietal (d) activities during 130–150ms and 190–220ms after the test onset respectively. These timings correspond to averaged frontal N150 and parietal N200 peak latencies. Blue and red lines correspond to whole report (WR) and partial report (PR) trials, respectively. Pink bar in both graphs represents test stimulus presentation time. Gray areas depict the period with a significant difference between ERP area under curve of WR and PR trials (*p*<0.05).

Finding neural correlates underlying filtering of irrelevant objects from WM we applied several two-factor ANOVAs (trial type and sample objects number) to ERP area under curve, recorded from frontal, parietal, occipital and temporal leads, across 800ms after the test stimuli onset (see method). Using this method we were able to examine all time intervals without any bias toward predefined ERP components (e.g. P1, N1 or P3). This measure enabled us to find any possible effect of irrelevant objects elimination (i.e. trials type effect) and memory load (i.e. sample objects number) through ERP activities in various brain regions. Using this method we found that in both frontal (figure 4a) and parietal (figure 4b) leads, ERP activities in response to partial report (PR) trials, which needed irrelevancy elimination, dissociated from ERP activities in response to whole report (WR) trials, which did not require any irrelevancy elimination. However, the onset time of this dissociation varied largely between these two cortical areas. The first site that showed significantly dissociable ERP activities was frontal area (two-factor ANOVA (sample objects number vs. trials type); F(1,132) = 5.247, *p*<0.05). In frontal leads this dissociation started from 152ms after the test onset but in parietal leads, the first significant dissociation (F(1,132) = 12.828, *p*<0.01) occurred 208ms after test onset which was 56 later than the same effect found in frontal leads. In both sites, similar to participants' RT, WR trials showed earlier rise of ERP signal compared to PR trials (see figure 4). Except for this rise time difference, in both sites the pattern of ERP signal (positive-negative-positive) remained intact during both types of trials.

We also checked whether sample set size or the number of relevant objects, besides the trial type, affected the frontal and parietal ERP activities or not. We did not find any significant effect of number of sample objects or interaction (F(5,132)<1, *p*>0.05) between the sample objects number and trials type in the first 400ms of ERP activities. Another sets of one-factor ANOVAs (test object number) and also two-factor ANOVA (test object number and trial types) did not yield any effect of relevancy load (i.e. test object number) or interaction between relevancy load and trial types on frontal and parietal ERP activities during first 400ms time window after the test onset (F(4,110)<1, *p*>0.05). While, the effect of trial types on both frontal and parietal ERPs remained significant in the later test (F(1,110)>5, *p*<0.05) similar to our previous tests mentioned in previous paragraph. Thus, early ERP activities (first 400ms) were only sensitive to the trials type and not the sample set size or the number of relevant objects number.

**Figure 5:**
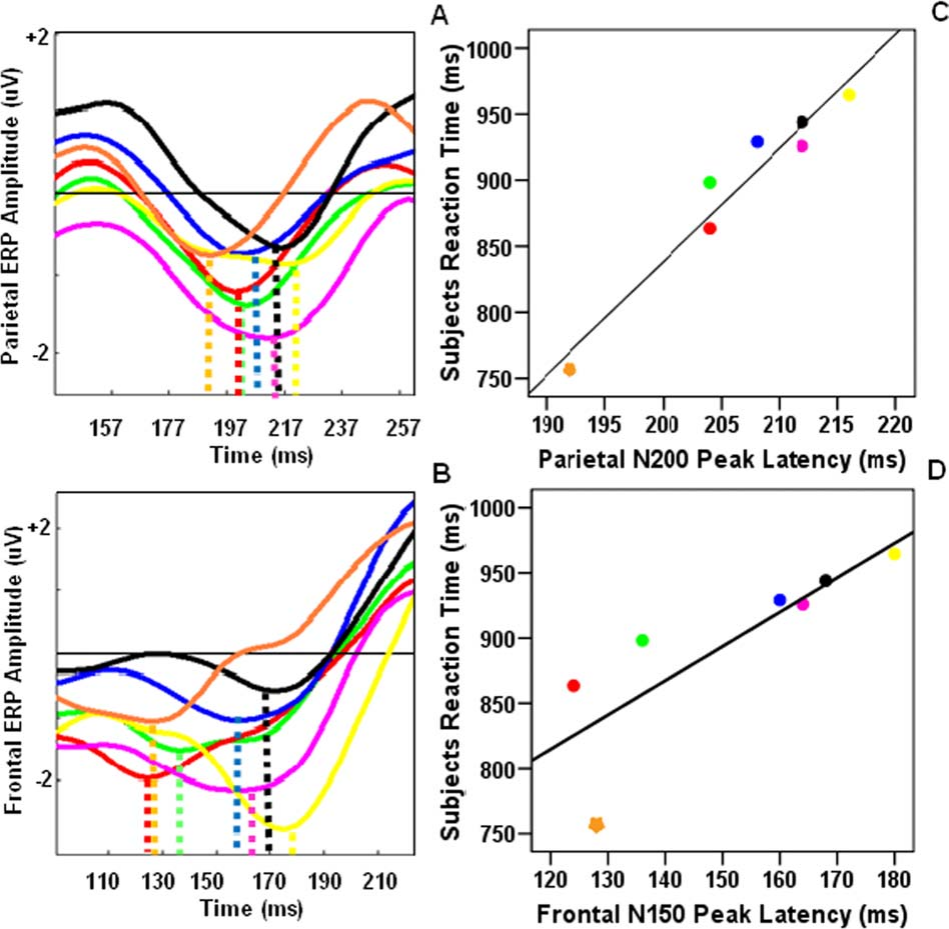
The relation between the parietal N200 (a, c) and frontal N150 (b, d) peak latencies and the participants' RT. Plots represent the ERP activities (left) and the corresponding scatter plots of their peak times (right). In both brain regions the peak latency of the ERP components increased with increasing the number of irrelevant objects and correlated with participants' RT. In scatter plots asterisks demonstrate values corresponding to whole report (WR) trials and squares depict values of partial report (PR) trials. The numbers close to each square show the amount of irrelevant load in each PR trial. Colour legends are the same for the scatter plots and the ERP plot. Lines in part c and d demonstrate the regression line.

We further examined whether the shift of ERP latency between PR and WR trials, reported above, could be also observed during different PR trials with different number of irrelevant objects. We assessed the latency of ERP negative peak, adjacent to the area that the ERP in response to PR trials dissociated from ERP in response to WR trials (i.e. N150 and N200 in frontal and parietal cortices, respectively). Brain activity mapping in 130–170ms and 190–230ms intervals after test onset suggested that N150 and N200 activities were localized in frontal and parietal areas, respectively (figure 4b, d). Assessing peak latency of these negative ERP components we found that, similar to participants' RT, frontal N150 and parietal N200 peak latencies varied with the amount of irrelevancy load. But, in parietal leads, rather than being linearly correlated to the irrelevancy load, about 46% of latency variation was related to the difference between WR and the PR trials with the minimum amount of irrelevancy load and shortest RT (Figure 5a, c). In spite of the short range of parietal N200 latency variation (25ms), this characteristic of parietal N200 activity was highly similar to participants' RT because 50% of participants' RT variation was also related to the difference between WR and fastest PR trials (see Figure 3b). Pearson test of correlation yielded a significant correlation between participants' RT and peak latency of parietal N200 activity (r = 0.830, *p*<0.05). In contrast to parietal N200 latencies, frontal N150 latency better encoded the amount of irrelevancy load in each trial and varied linearly with the number of irrelevant objects (r = 0.847, *p*<0.05). It also showed greater range of variation (55ms) between trials with different irrelevant object number (Figure 5b, d).

We further ruled out any possibility that the difference between WR and PR related activities was due to base-line differences between two experimental conditions. As we mentioned before in the method section, base-line was corrected on the basis of 100ms pre-sample activities rather than pre-test activities. If baseline variation, before test stimulus onset, was responsible for the difference between PR and WR activities, we expected to see significant correlation between frontal and parietal ERP amplitudes and participants RT. Two separate applications of Pearson tests of correlation did not yield any significant correlation between the ERPs amplitude and participants RT (*p*>0.05). It is noteworthy that base-line correction according to pre-test activities, rather than pre-sample activities, did not affect our findings. We avoided using this method of base-line correction since pre-test activities did not correctly represent the baseline and could be contaminated with those activities related to sample retention. Thus, frontal N150 and parietal N200 peak latencies, and not their peak amplitude, varied with irrelevancy load and pre-test base-line differences could not be responsible for this correlation.

**Figure 6:**
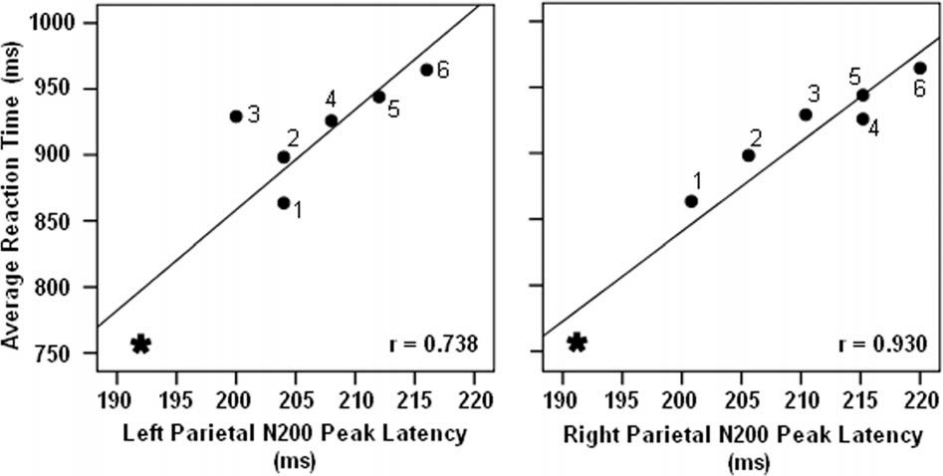
The relation between the parietal N200 peak latencies recorded in left (a) and right (b) hemispheres and the participants' RT. Parietal N200 peak latency in right hemisphere showed better correlation to participants' RT. Each symbol depicts values related to whole report (asterisk) and partial report trials (square) and the lines regression line.

Since previous studies have indicated a contra-lateral organization for memory retention [5]–[6] and right hemisphere dominancy [14] for change detection we checked whether noise filtering process was also lateralized or not. We found that parietal N200 peak latency in right hemisphere seems to be better correlated to participants' RT (r = 0.930, *p*<0.001) compared to the left hemisphere (r = 0.738, *p*<0.05) (Figure 6). Such difference was not found when we compared the amount of correlation between participants' RT and parietal N200 peak latencies within ipsi-lateral (r = 0.902, *p*<0.01) and contra-lateral (r = 0.881, *p*<0.01) hemispheres. In contrast to parietal N200 activity, comparing the correlation between frontal N150 activity and participants' RT (or irrelevancy load) within right (r = 0.881, *p*<0.05), left (r = 0.764, *p*<0.05), ipsi-lateral (r = 0.788, *p*<0.05) and contra-lateral (r = 0.799, *p*<0.05) hemispheres did not show any noticeable differences.

**Figure 7:**
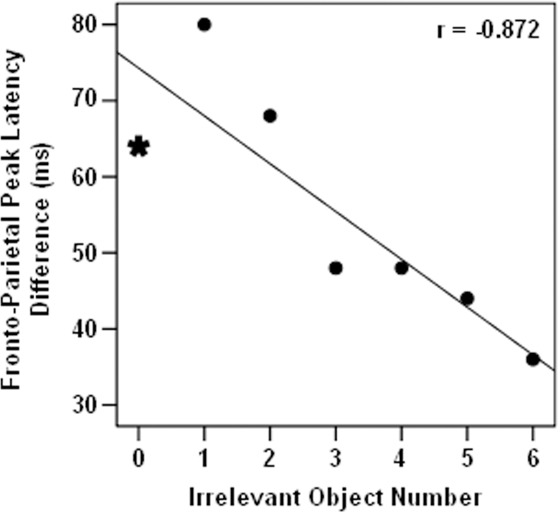
The difference between peak latencies of frontal N150 and parietal N200. The interval between the latency of these two ERP components decreased when the number of irrelevant objects increased (Pearson test of correlation, r = −0.872, *p* = 0.01). Symbols and lines represent are similar to figure 6.

To check whether functional connectivity [15]–[18] between modules generating ERP activities within frontal and parietal areas also varied with irrelevancy load, we assessed frontal N150 and parietal N200 latency differences. Interestingly, we found that frontal N150 and parietal N200 latency difference decreased as the number of irrelevant objects increased (Pearson correlation, r = −0.872, *p* = 0.01) indicating more strongly coupled activity in larger irrelevancy loads (Figure 7). We checked this relation in the left and right hemispheres separately and found that fronto-parietal ERP latency difference within the right hemisphere was better correlated with the amount of irrelevancy load (r = −0.906, *p*<0.01) compared to the fronto-parietal ERP latency difference within the left hemisphere (r = −0.762, *p*<0.05). This finding supports the notion that modules underlying ERP activities within right parietal area played a more crucial role in irrelevancy filtering process.

One factor that could have contaminated our results was unbalanced number of trials between PR (77.8% of all trials) and WR trials (22.2% of all trials). To check whether our findings emanated from unbalanced trial number between PR and WR trials we repeated the above mentioned analysis for WR trials against those trials with only one object in the test rather than all of PR trials. Here, the number of trials was identical between PR and WR trials. We found again a significant effect of trial type in both sites (*p*<0.05) and frontal activities showed earlier effect of trial type (t = 152ms after test onset) compared to the parietal activities (t = 212ms after test onset). The rest of analyses also showed the same tendency despite the fact that in this condition signal to noise ratio decreased to great extent.

## Discussion

In brief, here we have found that participants' RT was increased as we eliminated irrelevant objects between sample and test sets indicating that filtering mechanism rather than selection was used for eliminating irrelevant objects from WM. Similar to participants' RT, latency of ERP negativity in frontal (N150) and parietal (N200) cortices increased concomitant with the amount of irrelevancy load. In this condition, frontal N150 latency varied linearly with the number of irrelevant objects while parietal N200 latency was better correlated to participants' RT. This correlation was even stronger when we confined our analysis to right parietal activities, regardless of whether the memorized set was in ipsi or contra lateral hemisphere. We also found that the time difference between frontal N150 and parietal N200 latencies decreased as the irrelevancy load increased pointing to the notion that functional connectivity [15]–[18] between modules underlying these activities could vary with the amount of irrelevancy load.

Our behavioural findings were unlikely to be confounded by memory load (sample set size) or relevancy load (test set size) since we showed that participants' RT was significantly better correlated to the amount of irrelevancy load compared to memory or relevancy loads. Application of different ANOVAs to trials subsets with independent number of relevant and irrelevant objects also yielded that, our results was not confounded by dependency between relevancy and irrelevancy loads. It rules out the possibility that attentional shift between test objects or sample reconstruction is responsible for increased RT in PR trials. Rather it seems that detecting irrelevant objects and their suppression is responsible for delayed responses in PR report trials relative to WR trials.

Similar to participants' RT, frontal and parietal potentials in first 400ms after the test onset were also insensitive to the memory and relevancy load variations. Sweeping all time windows in this interval by using a sliding window method, confirmed that the only parameter that influenced frontal N150 and parietal N200 peak latencies was the amount of irrelevancy load. Thus, participants' RT and brain activities in a visual change detection task are highly under the influence of irrelevancy load, leaving only participants response accuracy and late parietal potential (not reported here) to be affected by sample objects number (memory load).

Parietal N200, reported in our study, differed from memory related activities reported by Vogel and Machizawa (2004). These authors' findings about memory related activities are confined to retention interval. These activities are measured by subtracting ipsi-lateral activities from the contra lateral ERPs within a fix interval (300–900ms after the sample onset) and are highly correlated to the number of objects within WM. But in our study, the parietal N200 was detected during decision making phase (after the test onset) and showed variable peak times that were correlated to subjects' RT. Furthermore, rather than being localized in contra lateral hemisphere relative to the memorized sets, parietal N200 was observed in both hemispheres with relatively stronger correlation between subjects' RT and right parietal N200 compared to the left parietal N200. Parietal N200 also varied from N2pc component which is mainly detected in visual search tasks whenever subjects attend to target object or its location [19]–[20]. Similar to memory related activities, this component is detected in contra lateral hemisphere relative to the attended hemifield without any correlation between its onset time and subjects' RT. High correlation between subjects RT and parietal N200 rules out the possibility that parietal N200 and N2pc component are generated by the same modules or represent the same phenomenon as described in our study.

Imaging studies have shown that frontal activity is significantly higher in trials with successful elimination of distracter interference on participants' memory performance [1]–[2]. We found that frontal N150 peak latency was better correlated with the number of irrelevant objects and not participants' RT and, presumably, attentional demand [21] of irrelevancy filtering. Our observation of the effect of irrelevancy load, besides the lack of any significant effect of sample or test sizes on frontal activities, indicates that module(s) underlying frontal activity is also a part of network which participates in irrelevancy filtering when the irrelevant objects are already stored in WM. However, being linearly correlated to the number of irrelevant objects, rather than participants' RT raises the possibility that frontal activity is related to irrelevant objects detection rather than their elimination. Contribution of frontal cortex in detection of irrelevant objects is previously indicated by fMRI imaging studies [22]–[23]. Interestingly, this frontal cortex contribution was not limited to one specific type of search suggesting a critical role for this brain area during different types of visual search tasks.

Importantly, here we found that in addition to frontal N150, parietal N200 was tightly correlated with the task irrelevancy load indicating that modules underlying parietal N200 activity could be a part of the irrelevancy filtering network. At first glance this finding seems to be in contrast to the previous studies which have not found any evidence for parietal involvement in filtering of irrelevant objects [1]–[3]. This inconsistency could be related to the difference between the natures of the tasks used in these studies. In these studies irrelevant objects are defined from the beginning of each trial and filtering role is confined to avoiding the irrelevant objects from entering WM. But in our study, all of the objects presented in the sample set could be relevant and had to be stored in WM and the irrelevancy of objects was revealed when the test set was presented. Thus, in our study, irrelevant objects needed to be excluded from WM and further decision related processes in a later stage of the task. Therefore, parietal N200 might be the neural correlate of the elimination of irrelevant objects from WM.

Since in our study the number of relevant and irrelevant objects could not be manipulated completely independent from each other, it seems plausible that the observed increase in RTs and ERP component latencies could still be due to a prolonged search for the test objects in WM. This hypothesis would be consistent with the involvement of the parietal cortex in selection within working memory representations [24]–[25]. However, our behavioural results suggest that increasing the number of relevant objects could not be responsible for delayed subjects' RT. As we showed that there was not any significant effect of relevancy load in the subset of trials with completely independent number of relevant and irrelevant objects. Thus it is more likely that filtering of irrelevant objects rather than selection of relevant ones was responsible for our subjects delayed RTs. Our ERP results also expand previous findings by suggesting two different modules to be responsible for irrelevant object elimination from WM whose connectivity increased concomitant with irrelevancy load. Our finding that there was no significant effect of number of relevant objects on frontal and parietal ERPs strengthens the possibility that filtering and not selection is used for elimination of irrelevant objects from WM.

In this experiment we also assessed the mechanism of interaction between modules underlying frontal N150 and parietal N200 ERP activities. Here we showed that frontal ERP potentials related to irrelevancy elimination, on average, starts 50ms earlier than the parietal potentials. The temporal lead of frontal activities relative to the parietal ones in demonstration of irrelevancy elimination effect raises the possibility that elimination related activities within parietal leads is initiated by modules underlying frontal N150. Findings from different lines of experiments support this notion. For example, neuroanatomical studies have shown that frontal afferent connections trigger inhibition mechanisms within temporal cortex, presumably initiating a distracter suppression process [26]–[27]. Others have shown that frontal cortex controls the impact of distracters when cortical sensory areas are exposed to noisy environments [28]–[29]. Since previous WM studies have shown that parietal cortex is responsible for retaining objects representations in WM [4]–[5] the parietal N200 potential in our study could be related to processes responsible for suppressing the irrelevant objects representation from WM. This hypothesis rely on the idea that frontal control is not limited to sensory areas and it also covers memory related areas such as parietal cortex. Heavy reciprocal connections between these two cortical areas could provide the necessary neural substrate for such interaction [30]–[31]. This idea was also supported by the enhanced connectivity, between modules underlying these ERP activities, with increasing irrelevancy load.

Here we presented evidence that the difference between frontal N150 and parietal N200 peak latencies decreased by increasing the amount of irrelevancy load. This variation could be related to the enhanced functional connectivity between the modules underlying frontal N150 and parietal N200 activities as irrelevancy load was increased. The enhancement could result in faster rising of parietal N200 relative to frontal N150 activity and therefore shorter intervals between the peaks of these two potentials. This idea is supported by previous studies showing that increasing task difficulty or attentional demand could activate new functional connections between frontal and parietal cortices which facilitate frontal access to parietal WM resources [17]–[18]. Similarly here, we showed that filtering processes highly relies on fronto-parietal ERP activities. Since filtering could be initiated and probably controlled by modules underlying frontal ERP activities, enhanced connectivity between modules underlying frontal and parietal potentials might be necessary in higher irrelevancy loads.

Comparing left and right parietal activities showed that in general right parietal activity seems to play a more crucial role in the irrelevancy elimination. Parietal N200 activity within right hemisphere was better correlated with the participants' RT. Interestingly, it also demonstrated more systematic fronto-parietal connectivity enhancement when irrelevancy load increased which points to the notion that it is more under the influence of task demand. This lateralized effect is consistent with previous study by Beck et *al.* (2006) showing that, in a change detection task, stimulation of right parietal, but not the left, cortex results in longer participants' RTs compared to non-stimulated conditions. On the basis of these findings we suggest that modules underlying right parietal N200 could play a more crucial role in irrelevancy elimination despite the fact that previous studies [5]–[6] have shown that contra lateral parietal activities, and not only right hemisphere, are correlated to objects maintenance in WM. This inconsistency could be related to different neural mechanisms responsible for memory retention and irrelevancy elimination within parietal cortex.

In conclusion, we showed a sequence of fronto-parietal ERP activities to be responsible for irrelevancy filtering when relevant and irrelevant information are stored in WM. Although we could not be sure about the location of modules underlying these ERP potentials, we showed great similarities between frontal and parietal activities reported by previous imaging studies and fronto-parietal ERPs found in our experiment. While imaging studies suffer from poor temporal resolution, and consequently miss the dynamics of neural activities, our results provide direct evidences that functional connectivity between modules underlying these fronto-parietal ERP activities was enhanced correlated with the irrelevancy load. This connectivity enhancement could facilitate the fronto-parietal interaction as the filtering demand increased.
